# Integrating Acute Polytrauma Rehabilitation Into Modern Trauma Systems: Benefits, Challenges, and Future Directions

**DOI:** 10.7759/cureus.103692

**Published:** 2026-02-16

**Authors:** Mohammed Tanvir Shah, Shuheda K Shah, Muhammed Monjur Ahmed

**Affiliations:** 1 Trauma and Orthopaedics, Royal London Hospital, Barts Health NHS Trust, London, GBR; 2 Internal Medicine, Southend University Hospital, Southend-on-Sea, GBR

**Keywords:** functional recovery, major trauma, polytrauma, rehabilitation, trauma systems, uk

## Abstract

Major trauma survival has improved substantially following the development of regional trauma systems. However, long-term disability among survivors remains common. Acute polytrauma rehabilitation, defined as a structured, multidisciplinary intervention initiated during the early inpatient phase following injury, is increasingly recognised as a key determinant of functional recovery, psychological health, and community reintegration. Despite growing international evidence, implementation within the United Kingdom (UK) Major Trauma Network remains inconsistent due to workforce constraints, commissioning fragmentation, and digital discontinuity. This review synthesises physiological, clinical, and system-level evidence supporting early rehabilitation; compares international trauma rehabilitation models; and critically examines barriers to implementation within the UK. Evidence-based recommendations are proposed to strengthen workforce capacity, data integration, and commissioning alignment. Embedding rehabilitation as a core pillar of trauma care is essential to ensuring that improved survival translates into meaningful long-term recovery.

## Introduction and background

Major trauma remains a leading cause of morbidity and premature mortality worldwide [1]. Advances in organised trauma systems, including regionalisation of care and network-based service delivery, have improved survival rates across high-income countries [2]. In England, the establishment of the Major Trauma Network in 2012 was associated with significant reductions in mortality following severe injury [3]. However, improved survival has revealed a substantial burden of long-term disability, with many survivors experiencing persistent physical, cognitive, and psychological impairment at one year post-injury [4].

Acute polytrauma rehabilitation refers to structured multidisciplinary rehabilitation initiated during the acute inpatient phase following major trauma, with early assessment and intervention recommended within the first days of hospital admission [5]. This model integrates physiotherapy, occupational therapy, speech and language therapy, clinical psychology, and specialist rehabilitation medicine alongside acute surgical and critical care management. National Institute for Health and Care Excellence guidance [5], Healthcare Quality Improvement Partnership audit data [6], and the World Health Organization Rehabilitation 2030 initiative [7] all recognise early rehabilitation as an essential component of trauma care delivery.

Despite this consensus, rehabilitation provision across United Kingdom (UK) trauma systems remains variable. Differences in workforce capacity, commissioning structures, and digital interoperability continue to influence access and intensity of rehabilitation services [6,8]. This review examines the physiological rationale for early intervention, evaluates international models of rehabilitation integration within trauma systems, and identifies structural barriers limiting consistent implementation.

## Review

Methodology

A narrative review methodology was employed to synthesise clinical, physiological, and system-level evidence relating to acute polytrauma rehabilitation. This approach allows integration of heterogeneous evidence sources, including observational cohorts, registry analyses, economic evaluations, and policy documents, where quantitative meta-analysis would not be methodologically appropriate [9].

A structured literature search was conducted using PubMed, Embase, CINAHL, Google Scholar, and the Cochrane Library between January 2005 and January 2026. Search terms included major trauma, polytrauma, early rehabilitation, acute rehabilitation, multidisciplinary therapy, functional outcomes, trauma systems, and return to work. Reference lists of included studies were hand-searched for additional literature.

Grey literature was identified through Healthcare Quality Improvement Partnership publications, national guideline documents, and international trauma registry outputs [5-7]. Eligible sources included systematic reviews, cohort studies, registry analyses, qualitative studies, policy documents, and economic evaluations addressing early rehabilitation following adult major trauma. Studies focusing exclusively on elective rehabilitation or paediatric-only populations were excluded unless providing relevant organisational insights.

Study selection and thematic synthesis were undertaken by a single author. Given the narrative design and inclusion of heterogeneous evidence types, a formal risk-of-bias assessment was not performed. Reporting principles were informed by guidance for narrative and scoping reviews [9].

Physiological rationale for early rehabilitation

Prolonged immobilisation following trauma results in rapid skeletal muscle atrophy and neuromuscular weakness, particularly in critically ill populations [10]. Experimental and clinical evidence demonstrates structural muscle changes and connective tissue adaptations during periods of inactivity within the first week of bed rest [11]. Joint immobility accelerates capsular fibrosis and contracture formation, limiting later recovery if early mobilisation is delayed [12].

Cardiopulmonary deconditioning also occurs rapidly during bed rest. Inactivity increases the risk of atelectasis, pneumonia, and venous thromboembolism [13]. Trauma-specific early mobilisation protocols in intensive care settings have demonstrated feasibility and associations with improved mobility outcomes [14].

Neurologically, early sensory and motor stimulation supports neuroplastic adaptation following traumatic brain injury and spinal cord injury. Longitudinal trauma cohort studies have demonstrated associations between early coordinated rehabilitation and improved functional recovery trajectories [4,15]. Speech and language therapy input is particularly relevant following severe traumatic brain injury, where dysphagia and communication impairment are common and influence morbidity [16].

Psychological engagement during the acute phase is also clinically significant. Systematic reviews have shown that early structured psychological intervention following trauma exposure may reduce post-traumatic stress symptoms and improve psychological outcomes [17].

Collectively, these mechanisms support integration of rehabilitation alongside acute surgical and critical care management rather than deferring intervention until later stages of recovery.

Evidence for early integrated rehabilitation

International trauma registries and observational cohorts indicate that early multidisciplinary rehabilitation is associated with improved functional outcomes following major trauma [4,15]. Rehabilitation initiated during the early inpatient phase has been linked to higher Functional Independence Measure scores at discharge and reduced hospital length of stay [18].

Longitudinal data demonstrate improved return-to-work rates and enhanced health-related quality of life among patients receiving coordinated rehabilitation input [19]. System-level analyses further suggest that timely rehabilitation reduces downstream healthcare utilisation and improves patient flow across trauma pathways [8]. Table 1 presents representative studies evaluating early rehabilitation outcomes in major trauma.

**Table 1:** Representative studies evaluating early rehabilitation outcomes in major trauma.

Study	Setting	Key findings
Lussiez et al. [8]	United States	Hospital-level factors drive variation in rehabilitation access
Klang et al. [15]	Sweden	National access to rehabilitation affects recovery trajectories
Jones et al. [18]	United Kingdom	Rehabilitation provision and geography influence outcomes
Kimmel et al. [20]	Australia	Intensive allied health therapy is associated with improved outcomes

Although causality cannot be definitively established due to observational study design, consistency across international datasets supports early integration of rehabilitation within trauma systems.

Domains of acute polytrauma rehabilitation

Physiotherapy targets restoration of strength, mobility, and cardiopulmonary function while mitigating deconditioning-related complications [13,14]. Occupational therapy focuses on activities of daily living, cognitive assessment, and discharge planning to facilitate functional independence [18].

Speech and language therapy plays a critical role following traumatic brain injury, where dysphagia and communication impairment are prevalent [16]. Psychological rehabilitation supports adjustment and reduces post-traumatic stress symptoms following major injury [17].

These domains operate synergistically within a multidisciplinary framework, aligning biological recovery with functional reintegration.

International trauma rehabilitation models

Germany’s TraumaNetzwerk DGU incorporates structured rehabilitation planning within trauma pathways, promoting continuity from acute care to reintegration [21]. Australia’s trauma system integrates allied health services early within acute care and links registry outcomes to performance monitoring [20]. Sweden’s national datasets enable evaluation of rehabilitation access and functional recovery trajectories [15]. In the United States, hospital-level and commissioning factors influence rehabilitation access and variability in post-acute care delivery [8]. These models demonstrate that effective integration requires coordinated governance, workforce investment, and digital infrastructure.

Challenges to implementation

Workforce shortages remain a primary barrier to consistent early rehabilitation delivery within UK trauma systems [6]. Variation in rehabilitation prescription implementation across trauma centres has been documented nationally [22]. Fragmented commissioning and inconsistent digital interoperability further impair care continuity [6,22]. Addressing these barriers requires structural reform and system-level alignment rather than isolated service adjustments.

Future directions

Future trauma system development must embed rehabilitation as a core pillar of care. Priorities include expanding rehabilitation workforce capacity [6], linking outcome registries to trauma audit datasets [22], and implementing interoperable electronic rehabilitation prescriptions [22]. Commissioning reform should align funding across the trauma pathway, reducing fragmentation between acute, specialist, and community services [6].

Limitations

This review has limitations inherent to narrative synthesis. Study identification and thematic analysis were performed by a single author, and no formal risk-of-bias assessment was undertaken. The evidence base remains predominantly observational, limiting causal inference. Variation in intervention timing, intensity, and outcome measurement across studies may influence generalisability. Further prospective trauma-specific interventional research is required.

## Conclusions

Acute polytrauma rehabilitation is fundamental to achieving meaningful recovery following major trauma. Early multidisciplinary intervention supports physiological stability, enhances functional recovery, and improves long-term participation outcomes. Strengthening rehabilitation integration within trauma systems is essential to ensure that survival gains translate into sustained quality of life.

## References

[1] (2020). Global burden of 369 diseases and injuries in 204 countries and territories, 1990-2019: a systematic analysis for the Global Burden of Disease Study 2019. Lancet.

[2] Cameron PA, Gabbe BJ, Cooper DJ, Walker T, Judson R, McNeil J (2008). A statewide system of trauma care in Victoria: effect on patient survival. Med J Aust.

[3] Moran CG, Lecky F, Bouamra O (2018). Changing the system - major trauma patients and their outcomes in the NHS (England) 2008-17. EClinicalMedicine.

[4] Gabbe BJ, Simpson PM, Harrison JE (2016). Return to work and functional outcomes after major trauma: who recovers, when, and how well?. Ann Surg.

[5] (2025). National Institute for Health and Care Excellence (NICE). NG211: Rehabilitation after traumatic injury. http://www.nice.org.uk/guidance/ng211.

[6] Healthcare Quality Improvement Partnership (HQIP) (2016). National Clinical Audit of Specialist Rehabilitation following Major Injury (NCASRI). London.

[7] (2025). World Health Organization. Rehabilitation 2030: A call for action. http://www.who.int/publications/m/item/rehabilitation-2030-a-call-for-action.

[8] Lussiez A, Montgomery JR, Sangji NF (2021). Hospital effects drive variation in access to inpatient rehabilitation after trauma. J Trauma Acute Care Surg.

[9] Tricco AC, Lillie E, Zarin W (2018). PRISMA Extension for Scoping Reviews (PRISMA-ScR): checklist and explanation. Ann Intern Med.

[10] Needham DM, Korupolu R, Zanni JM (2010). Early physical medicine and rehabilitation for patients with acute respiratory failure: a quality improvement project. Arch Phys Med Rehabil.

[11] Lad H, Saumur TM, Herridge MS, Dos Santos CC, Mathur S, Batt J, Gilbert PM (2020). Intensive care unit-acquired weakness: not just another muscle atrophying condition. Int J Mol Sci.

[12] Clavet H, Hébert PC, Fergusson D, Doucette S, Trudel G (2008). Joint contracture following prolonged stay in the intensive care unit. CMAJ.

[13] Alaparthi GK, Gatty A, Samuel SR, Amaravadi SK (2020). Effectiveness, safety, and barriers to early mobilization in the intensive care unit. Crit Care Res Pract.

[14] Coles SJ, Erdogan M, Higgins SD, Green RS (2020). Impact of an early mobilization protocol on outcomes in trauma patients admitted to the intensive care unit: a retrospective pre-post study. J Trauma Acute Care Surg.

[15] Klang A, Molero Y, Lichtenstein P (2023). Access to rehabilitation after hospitalization for traumatic brain injury: a national longitudinal cohort study in Sweden. Neurorehabil Neural Repair.

[16] Lee WK, Yeom J, Lee WH, Seo HG, Oh BM, Han TR (2016). Characteristics of dysphagia in severe traumatic brain injury patients: a comparison with stroke patients. Ann Rehabil Med.

[17] Roberts NP, Kitchiner NJ, Kenardy J, Lewis CE, Bisson JI (2019). Early psychological intervention following recent trauma: a systematic review and meta-analysis. Eur J Psychotraumatol.

[18] Jones SM, West C, Rappoport J, Akhtar K (2023). Rehabilitation outcomes based on service provision and geographical location for patients with multiple trauma: a mixed-method systematic review. Injury.

[19] Haider AH, Herrera-Escobar JP, Al Rafai SS (2020). Factors associated with long-term outcomes after injury: results of the Functional Outcomes and Recovery After Trauma Emergencies (FORTE) multicenter cohort study. Ann Surg.

[20] Kimmel L, Webb M, McCaskie D, Maric V, Fitzgerald M, Gabbe B (2025). Outcomes following intensive allied health therapy in the acute hospital for trauma patients. Injury.

[21] Frink M, Kühne C, Debus F, Pries A, Ruchholtz S (2013). [The TraumaNetzwerk DGU project. Goals, conception, and successes achieved]. Unfallchirurg.

[22] Kettlewell J, Radford K, Timmons S (2024). What affects implementation of the UK major trauma rehabilitation prescription? A survey informed by the behaviour change wheel. Injury.

